# Helicobacter pylori as a Cause of Dyspepsia in Patients With End-Stage Renal Disease

**DOI:** 10.7759/cureus.83766

**Published:** 2025-05-09

**Authors:** Sajid Bhatti, Sidra German, Muhammad Aslam, Imran Ahmed, Ali Hyder, Khaild Tareen, Hina Ismail, Raja Taha Yaseen Khan, Nasir Hassan Luck

**Affiliations:** 1 Nephrology, Sindh Institute of Urology and Transplantation, Karachi, PAK; 2 Gastroenterology, Madina Teaching Hospital, Faisalabad, PAK; 3 General Internal Medicine, King’s College Hospital, London, GBR; 4 Gastroenterology, Chandka Medical College, Shaheed Mohtarma Benazir Bhutto Medical University, Larkana, PAK; 5 Gastroenterology and Hepatology, Bolan Medical Complex, Quetta, PAK; 6 Hepatogastroenterology, Sindh Institute of Urology and Transplantation, Karachi, PAK

**Keywords:** diabetes, dyspepsia, end-stage renal disease (esrd), helicobacter pylori, nonsteroidal anti-inflammatory drugs (nsaids)

## Abstract

Introduction

Dyspepsia is a frequent complaint in patients with end-stage renal disease (ESRD). *Helicobacter pylori* (*H. pylori*) is a well-established etiological factor for dyspepsia in the general population; however, its role in ESRD patients remains to be determined. Therefore, this study aimed to determine the frequency of *H. pylori* infection among ESRD patients presenting with dyspeptic symptoms.

Methodology

A cross-sectional study was conducted at the Sindh Institute of Urology and Transplantation from January 2023 to June 2024. A total of 200 adult ESRD patients undergoing maintenance hemodialysis and experiencing dyspepsia for at least three months were included. The patients with history of *H. pylori* infection or prior history of *H. pylori* eradication, those with history of usage of proton pump inhibitors or antibiotics or H2-receptor blockers within the past month, patients with gastric or duodenal ulcers, gastrointestinal malignancies or other systemic diseases causing dyspepsia and pregnant or breastfeeding females were excluded from the study. Upper gastrointestinal endoscopy and gastric biopsies were performed for histological confirmation of *H. pylori*. Data were analyzed using SPSS (IBM SPSS Statistics for Windows, IBM Corp., Version 27, Armonk, NY).

Results

Out of 200 patients, 80 (40%) were positive for *H. pylori*. Significant associations were observed between *H. pylori *infection and diabetes (p = 0.036), hypertension (p = 0.043), NSAID usage (p = 0.024), postprandial fullness (p ≤ 0.001), and epigastric pain (p = 0.041). Inversely, longer hemodialysis duration was associated with a lower prevalence of *H. pylori* (p = 0.012). Patients with *H. pylori* had lower hemoglobin and BMI levels and higher total leukocyte counts.

Conclusion

Our study showed that *H. pylori* is a common cause of dyspepsia in the ESRD population. Screening and eradication strategies may improve symptom control and enhance quality of life in this population. Multi-centered studies with larger sample sizes are not only required to validate our results but also to assess therapeutic outcomes.

## Introduction

Dyspepsia is described as a chronic or recurrent discomfort in the upper abdomen that significantly impairs the quality of life in patients [1]. The overall estimated prevalence of dyspepsia in the general population lies between 10 and 20%, with *Helicobacter pylori* (*H. pylori*) as one of the most common causes associated with it [2-4]. *H. pylori* is a Gram-negative spiral bacterium that colonizes the gastric mucosa and is associated with a spectrum of peptic diseases including peptic ulcers, chronic gastritis, and gastric cancer [5-7].

The role of *H. pylori* in dyspepsia has been extensively studied in the general population, but its implications in specific subgroups of patients, such as those with end-stage renal disease (ESRD), remain under-researched. Patients with ESRD require either dialysis or kidney transplantation for their survival [8]. Gastrointestinal symptoms, mainly dyspepsia, are common in ESRD patients, with the prevalence ranging between 30% and 70% of such patients [9]. Many factors are attributed to it, including delayed gastric emptying, the presence of uremic toxins, a changing gut microbiome, and the use of NSAIDs [10].

The exact role of *H. pylori* infection as a cause of dyspepsia in the ESRD population is uncertain. However, some studies showed a lower incidence due to a possible uremia-induced hostile gastric environment inhibiting *H. pylori* [11]. On the other side, there exist similar or slightly higher incidences of *H. pylori* in ESRD patients as compared to the general population [12].

In Pakistan, the overall infection rate of *H. pylori* is quite high, ranging between 50% and 90%, due to poor sanitation, overcrowding, and lack of access to health facilities [13-15]. However, data on its role in dyspepsia among ESRD patients in Pakistan are scant. This relationship is of utmost importance because dyspepsia adds significantly to the morbidity in ESRD patients, thus influencing their adherence to dialysis schedules and impacting overall quality of their lives. The role of *H. pylori* as a contributing modifiable variable could provide targeted therapeutic interventions aimed at improved symptom modulation and reduced costs of care, thus improving the overall quality of life of ESRD patients presenting with dyspepsia. Therefore, the primary aim of this study was to determine the frequency of *H. pylori* infection among ESRD patients presenting with symptoms of dyspepsia.

## Materials and methods

Study design

After the approval from the Sindh Institute of Urology and Transplantation Ethical Review Committee (approval number: SIUT-ERC-2022/A-312), this cross-sectional study was carried out at the Departments of Nephrology and Hepatogastroenterology, Sindh Institute of Urology and Transplantation from 1st January 2023 to 30th June 2024. It included all the patients of either gender aged 18 years or older undergoing maintenance hemodialysis and presenting with dyspeptic symptoms for at least three months. The patients with history of *H. pylori* infection or prior history of *H. pylori* eradication, those with history of usage of proton pump inhibitors or antibiotics or H2-receptor blockers within the past month, patients with gastric or duodenal ulcers, gastrointestinal malignancies, or other systemic diseases, causing dyspepsia and pregnant or breastfeeding females were excluded from the study.

Sampling technique and sample size 

The patients were enrolled using the non-probability consecutive sampling method. Considering the estimated prevalence of *H. pylori* as a cause of dyspepsia in the ESRD population as 40% (as no local data were available), a 95% confidence interval, and a margin of error of 5%, a sample size was set at 200 patients.

Data collection

After taking informed consent, patients undergoing maintenance hemodialysis and presenting with dyspeptic symptoms for at least three months were enrolled in the study. Demographic data, including the medical history, clinical symptoms, and baseline laboratory investigations, were recorded. The diagnosis of *H. pylori* was done by performing the upper gastrointestinal endoscopy and biopsies from the body and antrum of the stomach in each patient. For the detection of *H. pylori*, biopsy specimens were immersed in formalin and sent for histopathological examination by an expert histopathologist with more than 20 years of experience in gastrointestinal and infectious diseases.

Data analysis procedure

Data was entered and analyzed using SPSS (IBM SPSS Statistics for Windows, IBM Corp., Version 27, Armonk, NY). Continuous variables were expressed as mean ± standard deviation, while the expression of categorical variables was done in the form of frequencies and percentages. Outcome was recorded as the presence or absence of *H. pylori* infection on histopathology. Continuous variables were analyzed using the Student t-test, while categorical variables were analyzed using the chi-square tests for the presence or absence of *H. pylori*. A p-value of <0.05 was considered statistically significant.

## Results

A total of 200 patients with ESRD undergoing maintenance hemodialysis and presenting with dyspepsia were included in this study. Most of the patients (122 (61%)) were males. The mean age of the patients was 45 ± 9 years, and the mean duration of hemodialysis was 5 ± 1 years. The majority of patients had significant comorbidities, with hypertension present in 108 (54%) and diabetes mellitus present in 70 (35%) patients, respectively. Mean body mass index (BMI) was 22.3 ± 1.2 kg/m^2^. The history of NSAIDs usage was observed in 50 (25%) patients, while 44 (22%) patients were smokers.

Patients most commonly presented with the complaint of postprandial fullness that was observed in 136 (68%) patients, followed by epigastric pain in 112 (56%) patients, nausea in 72 (36%), bloating in 70 (35%), and early satiety in 32 (17%) patients, respectively. Mean hemoglobin was 11.1 ± 1.2 g/dL, total leucocyte count (TLC) was 4.2 ± 1.1 × 10^9^/L, platelet count was 261 ± 20 × 10^9^/L, and serum blood urea nitrogen (BUN) was 76.1 ± 14 mg/dL (Table 1).

**Table 1:** Baseline Characteristics of the population included in the study (n = 200) BUN - blood urea nitrogen; *H. pylori* - *Helicobacter pylori*; NSAID - nonsteroidal anti-inflammatory drug; TLC - total leucocyte count

Study population		n (%)
Gender	Males	122 (61)
	Females	78 (39)
Symptoms of dyspepsia at baseline	Postprandial fullness	136 (68)
	Epigastric pain	112 (56)
	Nausea	72 (36)
	Bloating	70 (35)
	Early satiety	32 (17)
Comorbidities	Hypertension	108 (54)
	Diabetes	70 (35)
*H. pylori* on histology	Present	80 (40)
	Absent	120 (60)
History of NSAID usage		50 (25)
History of smoking		44 (22)
Mean age (years)		45 ± 9
Duration of hemodialysis (years)		5 ± 1
Hemoglobin (g/dL)		11.1 ± 1.2
TLC (×10^9^/L)		4.2 ± 1.1
Platelet (×10^9^/L)		261 ± 20
BUN (mg/dL)		76.1 ± 14.0

On comparative analysis, history of NSAID usage (p = 0.024), presence of comorbidities like diabetes (p = 0.036) and hypertension (p = 0.043) along with symptoms of postprandial fullness (p ≤ 0.001) and epigastric pain (p = 0.041) were significantly more commonly in patients with *H. pylori* infection. Longer duration of hemodialysis (p = 0.012) was inversely related to the *H. pylori* infection. BMI (p = 0.048) and hemoglobin levels (p = 0.026) were also significantly lower, while TLC (p = 0.03) was significantly higher in patients with *H. pylori* infection (Table 2).

**Table 2:** Comparison of variables in predicting H. pylori gastritis (n = 200) *The chi-square test was used to calculate p-values of categorical variables. **Student t-test was used to calculate p-values of continuous variables. BUN - blood urea nitrogen; *H. pylori* - *Helicobacter pylori*; TLC - total leucocyte count

Variable		*H. pylori* infection		Chi-square value*/t-test value**	p-value
		Present (n = 80) N (%)	Absent (n = 120) N (%)		
Gender	Male	53 (66)	75 (62.5)	0.391	0.071
	Female	27 (34)	45 (37.5)		
Diabetes	Yes	52 (65)	18 (15)	0.659	0.036
	No	28 (35)	102 (85)		
Hypertension	Yes	66 (82.5)	42 (35)	0.421	0.043
	No	14 (17.5)	78 (65)		
Presenting complaints	Postprandial fullness	78 (97.5)	58 (48.3)	2.1	≤ 0.001
	Epigastric pain	77 (96.3)	35 (29.2)	1.7	0.041
	Nausea	28 (35)	44 (36.7)	0.043	0.593
	Bloating	19 (23.8)	51 (42.5)	0.067	0.08
	Early satiety	11 (13.8)	22 (18.3)	0.741	0.29
History of NSAID usage		28 (35)	22 (18.3)	10.898	0.024
Mean age (years ± SD)		43.5 ± 9.1	51.9 ± 7.5	-7.98	0.40
BMI (kg/m^2^)		20.4 ± 2.0	23.8 ± 1.2	-13.642	0.048
Duration of hemodialysis (years)		3.1 ± 1.4	8.4 ± 2.3	-5.951	0.012
Hemoglobin (g/dL)		9.8 ± 3.1	11.7 ± 2.2	-4.417	0.026
TLC (×10^9^/L)		6.4 ± 3.2	7.8 ± 1.4	-0.252	0.03
Platelet (×10^9^/L)		143 ± 84	213 ± 85	-3.025	0.09
BUN (mg/dL)		113 ± 41	101 ± 67	2.341	0.06

## Discussion

Our study aimed to identify the prevalence of *H. pylori* infection in patients with ESRD with dyspepsia, thus contributing to the ongoing debate regarding the role of *H. pylori* as a cause of dyspepsia in this vulnerable population. Several studies have previously examined this association with inconsistent results, depending on study design, study population, and diagnostic criteria.

In this study, we identified that *H. pylori* was present in 40% of ESRD patients with dyspepsia. This is in agreement with previous global reports but differs from some regional studies. A study by Shin et al. on Pakistan's general population reported a rate of *H. pylori* of 61.6% in 2011, which is significantly higher than our finding in ESRD patients [16]. Shin et al. reported a prevalence of *H. pylori* in CKD patients of about 48.2% as compared to 59% in the general population, consistent with that reported in this study [16]. Decreased rates in ESRD patients can be explained by the uremic environment that has been reported to suppress *H. pylori* colonization by elevated gastric pH and altered immune responses [17].

We found that diabetes and hypertension were significantly associated with *H. pylori* infection (p = 0.036 and p = 0.043, respectively). This is consistent with findings observed by Wang et al., who reported that diabetic ESRD patients are prone to gastric infections due to compromised immunity and alterations in gastric mucosal defense mechanisms [18]. Nevertheless, previously, no significant association was observed between diabetes and *H. pylori*, and it is postulated that diet and genetic susceptibility are involved.

In our study, we observed that *H. pylori* infection was significantly more common in patients with a history of NSAID use (p = 0.024). This is supported by findings by Sostres et al., who reported that NSAID-induced gastric mucosal injury can facilitate *H. pylori* colonization and increase dyspeptic symptoms [19]. We did not see any significant relation between smoking and *H. pylori* infection in our study, despite previous studies suggesting a possible relation.

Patients with a longer duration of hemodialysis (mean: 8.4 ± 2.3 years) were found to be less susceptible to *H. pylori* infection (p = 0.012). These findings are similar to those observed in the previous literature and can be related to the increased urea levels inhibiting the growth of *H. pylori* in the gastric mucosa in patients with ESRD [20,21].

Most common complaints in our study were postprandial fullness (97.5%) and epigastric pain (96.3%), and both were significantly correlated with *H. pylori* infection. Our findings are consistent with those of Wang et al., who reported that dyspeptic symptoms were increased in *H. pylori*-infected patients with ESRD [18]. Nausea, bloating, and early satiety did not show significant correlations and are likely to have multifactorial causes in patients with ESRD.

There are certain limitations that can be attributed to our study. First, the sample size of our patient population was limited to just 200 patients and may restrict generalizability. Second, we did not assess symptom resolution with eradication therapy for *H. pylori* in this study, and we may consider it for future studies. Larger studies with follow-up of symptom resolution after eradication therapy and a prospective design would provide more definitive results.

## Conclusions

This study emphasizes that *H. pylori* infection is a common and often underdiagnosed etiology of dyspeptic symptoms in patients with ESRD. The observed associations with certain clinical features and comorbidities suggest that multiple factors may influence the presence of infection. Diagnosis and targeted therapy of *H. pylori* in ESRD patients can not only improve the dyspeptic symptoms but can also improve the overall quality of life in this population. However, large-scale, prospectively designed studies with interventional arms to examine the effect of eradication therapy on clinical outcomes in this patient population are required to develop standardized treatment practices.
